# Methotrexate improves endothelial function in early rheumatoid arthritis patients after 3 months of treatment

**DOI:** 10.1186/s13075-022-02930-7

**Published:** 2022-10-24

**Authors:** Giacomo Cafaro, Eleonora Petito, Onelia Bistoni, Emanuela Falcinelli, Sabrina Cipriani, Maria Chiara Borghi, Angelo F. Bonifacio, Elisa Giglio, Alessia Alunno, Carlo Perricone, Roberto Gerli, Paolo Gresele, Elena Bartoloni

**Affiliations:** 1 Rheumatology Unit, University of Perugia, Perugia, Italy; 2 Section of Internal and Cardiovascular Medicine, Department of Medicine and Surgery, University of Perugia, Perugia, Italy; 3 Internal Medicine and Nephrology Unit, Department of Life, Health & Environmental Sciences, University of L’Aquila, L’Aquila, Italy

**Keywords:** Methotrexate, Rheumatoid arthritis, Endothelial dysfunction, Cardiovascular disease, Angiogenic T cells, Platelets, Microvesicles

## Abstract

**Background:**

Endothelial dysfunction contributes to increased cardiovascular (CV) disease in rheumatoid arthritis (RA). Angiogenic T cells (Tang) are a key regulator of vascular function via their interaction with endothelial progenitor cells (EPCs). Methotrexate (MTX) has been associated to reduced CV disease risk, but its effects on endothelial homeostasis have been poorly explored. We investigated MTX effects on endothelial homeostasis in early, treatment-naïve RA patients.

**Methods:**

Fifteen untreated, early RA patients and matched healthy controls (HC) were enrolled. RA patients with long-standing disease in remission or low disease activity treated with MTX for at least 6 months were selected as controls. Circulating CD28^+^ and CD28^null^ Tang cell, endothelial microparticle (EMP), EPC and soluble vascular cell adhesion molecule (sVCAM)-1 levels were measured.

**Results:**

Tang percentage was higher in early RA than in HCs and significantly increased after 3-month MTX treatment. Tang cells in RA were characterized by higher percentage of CD28^null^ and lower CD28-positive cells than HCs. MTX restored a Tang cell phenotype similar to HCs. Altered sVCAM-1, EMP and EPC were restored to levels similar to HCs after a 3-month MTX. Biomarker levels after 3 months of MTX were not different to those of patients with long-standing treatment.

**Conclusions:**

MTX has a positive effect on Tang, sVCAM-1, EPCs and EMPs in RA. Restoration of imbalance between CD28 + and CD28^null^ Tang by MTX may be one of the mechanisms underlying its favourable effects on endothelial dysfunction. These effects seem to be long-lasting and independent from systemic inflammation reduction, suggesting a direct effect of MTX on the endothelium.

## Background

Physiologic endothelial function is maintained thanks to a complex network of damage restoration mechanisms to which several molecules as well as cellular populations contribute [1]. Loss of endothelial layer integrity and function represents one of the earliest triggers of atherosclerosis and a pivotal mechanism contributing to the increased risk of cardiovascular (CV) disease in patients with chronic inflammatory disorders, including rheumatoid arthritis (RA) [2–8]. In this setting, inflammatory and immune-mediated mechanisms are still incompletely understood and interact with traditional CV risk factors in the induction and perpetuation of endothelial damage, thus contributing to CV risk [8, 9]. A key player of endothelial dysfunction is platelet CD40L, a type II transmembrane protein belonging to the tumour necrosis factor (TNF) superfamily expressed on the surface of activated platelets, which induces endothelial cell activation interacting with CD40, inducing an increase in vascular cell adhesion molecule-1 (VCAM-1) expression [10] and favouring atherosclerosis progression and increased CV risk [11–13]. In patients with systemic autoimmune diseases, an increase in circulating endothelial microparticles (EMPs) in association with progressive exhaustion of endothelial progenitor cells (EPCs) has been associated with endothelial damage and with defective vascular layer restoration [14, 15]. A specific T cell population, characterized by the co-expression of CD3, CD31 and CXCR4 and termed angiogenic T cells (Tang), has recently emerged as a key regulator of vascular function and neo-angiogenesis due to their interaction with EPCs [16]. Of note, altered levels of circulating Tang cells and of their senescent CD28^null^phenotype have been demonstrated to correlate with endothelial damage and adverse CV outcome in patients with systemic lupus erythematosus (SLE), RA and primary Sjögren’s syndrome (pSS) [17–20]. In these patients, as well as in subjects with increased CV risks such as hypertension and diabetes mellitus, Tang cells correlate with circulating EPCs and mainly exhibit an immunosenescent, pro-inflammatory and cytotoxic profile, suggesting their role as a reliable marker of accelerated atherosclerosis and endothelial dysfunction [17, 18, 21, 22].

The possible ability of drugs to improve these cellular biomarkers may help to identify new pharmacological strategies to prevent or reduce endothelial dysfunction. Methotrexate (MTX) represents the gold standard and first-line drug in the treatment of RA patients interfering with multiple inflammatory and metabolic pathways, such as homocysteine and nucleic acid metabolism [23]. Interestingly, long-term MTX therapy has been also associated with a 28% overall lower risk of major CV events, in particular, myocardial infarction, a 57% reduction of hospitalization for heart failure and with reduced CV mortality in RA patients [24, 25]. This probably reflects the reduction of the systemic inflammatory burden by MTX treatment, with a consequent decrease in atherosclerotic CV risk. Moreover, although with some conflicting evidence, improvement of arterial stiffness, endothelial dysfunction and carotid intima-media thickness, as well as of insulin resistance and lipid pro-atherogenic profile, has been observed in MTX-treated RA patients [26, 27]. In vitro and ex vivo studies, as well as in vivo experiments in mouse models of atherogenesis, have shown that MTX may exert direct anti-inflammatory and protective effects on endothelial layer and function. In particular, increased intracellular levels of adenosine by activation of adenosine monophosphate-protein kinase genes, reduction of inflammatory cytokines and oxidative stress, scavenging of free radicals and inhibition of neutrophil oxidative burst, as well as improvement of aortic thickening by acting on overexpression of endothelial cell beta-3 receptors, have been suggested as potential mechanisms in recent studies [28–32]. Interestingly, a significant reduction of serum syndeca-1 levels, reflecting an improvement of endothelial layer integrity, was observed following a 6-week MTX monotherapy in a small cohort of RA patients [33]. Taken together, these results suggest a protective, anti-inflammatory activity of MTX on endothelial layer integrity and function. However, the positive effect on endothelial homeostasis and the reduced risk of CV events in RA seem to be partly independent from MTX-induced improvement of disease activity, suggesting that alternative mechanisms may contribute to the improvement of endothelial function and reduction of CV risk [24, 33]. The effect of MTX on biomarkers and cell subsets of endothelial damage and repair has been poorly explored. In particular, no studies evaluated the behaviour of Tang and immunosenescent CD28^null^lymphocytes following MTX administration in patients with inflammatory rheumatic diseases. Thus, the aim of the present study was to investigate the effects of the 3-month MTX treatment on biomarkers of endothelial homeostasis in a cohort of treatment-naïve RA patients by evaluating the changes in circulating Tang cells and their subpopulations, sVCAM, PMPs, platelet CD40L, EPCs and CECs.

## Methods

### Patients

Consecutive, untreated, early (≤ 3 months from symptom onset) RA patients with moderately-highly active defined as Disease Activity Score on 28 Joints (DAS28) based on C-reactive protein (DAS28-CRP) ≥ 3.2 and age- and sex-matched healthy controls (HC) were enrolled between April 2018 and June 2019. A cohort of age- and sex-matched RA patients with long-standing disease in remission or low disease activity (DAS28 < 3.2) treated with MTX for at least 6 months was also selected as additional comparators. All RA patients fulfilled the 2010 ACR/EULAR classification criteria [34]. To avoid the risk of bias due to the presence of other factors with a potential influence on the markers under investigation, patients with a history of major CV disease (acute myocardial infarction, transient ischemic attacks, stroke, clinically relevant arrythmias, peripheral artery disease, congestive heart failure), taking anti-platelet or anti-coagulant agents, prednisone > 10 mg/die or who had taken non-steroidal anti-inflammatory drugs in the previous 7 days were excluded. No other disease-modifying anti-rheumatic drugs, nor hydroxychloroquine, were allowed. All patients underwent a detailed medical interview and clinical examination. For the purpose of the study, the following parameters were specifically collected: anthropometric measures (height, weight, body mass index), smoking status (current, former, never) and history of diabetes mellitus, dyslipidaemia and hypertension. Diabetes mellitus was defined by a fasting glucose of ≥ 126 mg/dL or the use of antidiabetic drugs. Hypertension was defined as a previous physician’s diagnosis or current anti-hypertensive treatment. Hypercholesterolaemia and hypertriglyceridaemia were defined as previous diagnosis or need for treatment as defined by the ESC/EAS Guidelines for the management of dyslipidaemias [35]. The 10-year risk of CV disease was calculated by the Q-RISK2 score as the Q-RISK3 score was not yet validated when the study was designed and approved [36]. Disease-related parameters were also recorded in all patients and included the following: tender joint count (TJC), swollen joint count (SJC), patient’s global assessment (PGA), physician’s global assessment (PhGA), CRP and erythrocyte sedimentation rate (ESR). Disease activity was calculated by DAS28 with both CRP and ESR, clinical disease activity index (CDAI) and simplified disease activity index (SDAI).

Study procedures were collected in early RA patients before and 3 months after the introduction of MTX treatment.

The study was conducted according to the guidelines of the Declaration of Helsinki. The protocol was approved by the local ethics committee *Comitato Etico Regionale Umbria*, 3110/17. All subjects provided written informed consent.

### Angiogenic T cells

Peripheral blood was collected in lithium-heparin tubes, and PBMCs were isolated by gradient using Lymphoprep (Stemcell Technologies, Vancouver, Canada) according to the manufacturer’s instructions. Staining with anti-CD4, anti-CD31, anti-CXCR4 and anti-CD28 antibodies (Becton, Dickinson and Company, NJ, USA) was performed, and data were acquired with BD FACSCalibur (Becton, Dickinson and Company, NJ, USA) flow cytometer and analysed with BD CellQuest Pro software (Becton, Dickinson and Company, NJ, USA).

### Endothelial activation markers

As endothelial activation markers, we assessed soluble VCAM-1 (sVCAM-1) by ELISA, endothelial-derived extracellular microvesicles (EVs), circulating endothelial cells (CECs) and circulating endothelial progenitor cells (EPCs) by flow cytometry, as previously reported [3, 4, 37].

Plasma levels of sVCAM-1 were measured by ELISA (R&D system, Abingdon, UK), as described [37]. The intraassay coefficient of variation for sVCAM-1 was 3.1%, and the interassay coefficient of variation was 7%. The results are reported as ng/mL.

Endothelial-derived extracellular microvesicles (EVs) were assessed by flow cytometry. Citrated plasma was centrifuged at 12,000 × *g* for 2 min to obtain platelet-free plasma (PFP). Fifty microlitres of PFP was incubated with an anti-CD146-PE-labelled antibody, an endothelial junctional cell adhesion molecule (CAM) belonging to the immunoglobulin superfamily, or with the isotype control antibody (all from Beckman Coulter, Miami, FL, USA). After 30 min at room temperature in the dark, the reaction was stopped with PBS. The microvesicle morphological gate was set using Megamix (BioCytex, Marseille, France), a mix of fluorescent beads of various diameters covering the microvesicle size range of 0.1 to 1 µm. To quantify EVs, 50 µL of a known concentration of Flow-Count Coulter fluorospheres (Beckman Coulter, Miami, FL, USA) was added to the FACS tubes prior to analysis. The results are reported as microvesicle number per microliter (EVs/μl) [4].

Circulating CECs and EPCs were identified and characterized in the whole blood collected in 0.18% K3EDTA by flow cytometry (Cytoflex, Beckman Coulter, Miami, FL, USA) using progenitor, haematopoietic and endothelial markers. For the identification of CECs, a combination of CD45, CD31 and CD146 was used, while for the identification of circulating EPCs, antibodies against human CD45, CD34 and CD309 (Beckman Coulter, Miami, FL, USA) were used. Briefly, 10 μL of each fluorochrome-labelled antibody was added to 100 μL of whole blood then incubated in the dark at room temperature for 30 min; 2 mL of red cell lysing solution was added, and the tube was incubated for 15 min at room temperature, prior to sample reading. To quantify cells, 100 μl of a known concentration of Flow Count Coulter fluorospheres was added to the FACS tubes just prior to analysis. The results were expressed as absolute cell number per microliter [4].

### Surface platelet activation markers

Venous whole blood was collected in trisodium citrate 3.2% (0.109 M, 1/10 v/v), and platelet CD40L was assessed by flow cytometry (CytoFLEX, Beckman Coulter, Miami, FL, USA) as previously reported [2]. Five microlitres of whole blood was incubated with saturating concentrations of anti-CD41 [platelet GPIIb]-fluorescein isothiocyanate (FITC)-labelled and anti-CD40L-phycoerythrin (PE)-labelled or isotype control antibody (IgG PE-labelled antibody) (all from Beckman Coulter, Miami, FL, USA). After 30 min of incubation in the dark, samples were diluted with PBS and analysed. The results are reported as the percentage of CD40L-positive platelets.

### Data analysis

Sample size calculation was not performed due to the absence of previous data and the pilot nature of the study. Continuous variables were analysed with the Wilcoxon signed-rank test (paired samples) or the Mann–Whitney *U* test (unpaired samples). Categorical variables were analysed with the *Χ*^2^ test or Fisher’s exact test, as appropriate. Correlations were analysed with the Spearman test. Data are considered significant for *p* ≤ 0.05. Samples with missing data were excluded sample-wise. All the analysis was performed with IBM SPSS Statistics v.26 (IBM Corp. Armonk, NY, USA). Plots were designed with GraphPad Prism version 8.0.0 (GraphPad Software, San Diego, CA, USA). Data are shown as mean ± standard error of the mean or absolute number and percentage.

## Results

### Participants’ characteristics

Fifteen patients with early RA and 15 age- and sex-matched HC were enrolled. At follow-up, one RA patient was excluded as treatment with acetylsalicylic acid was introduced after enrollment. Demographic features and traditional CV risk factors in both cohorts are shown in Table 1. Prevalence of current and former smoking habit and QRISK-2 score were significantly higher in RA patients as compared to HCs. The prevalence of hypertension, diabetes mellitus, hypercholesterolaemia and hypertriglyceridaemia was similar between the two groups. At baseline, RA patients were characterized by moderate-high disease activity according to SDAI, CDAI and DAS28-CRP scores. Disease activity significantly improved after 3 months of MTX treatment as shown by the DAS28-CRP and SDAI scores (Table 1).

**Table 1:** Characteristics of HC and RA patients at baseline and after 3 months of MTX treatment

	**Control group (HC),** ***n* = 15**	**Early RA (RA),** ***n* = 15**	***p* (HC vs RA)**	**MTX at 3 months (RA MTX T1),** ***n* = 14**	***p* (MTX 3 months vs RA)**
Age	53.3 ± 7.03	60 ± 8.06	0.25		
Sex (*n*, %F)	11 (73.3%)	11 (73.3%)	1.0		
Smoking habit			**0.038**		
Never smoker	11 (73.3%)	4 (26.7%)			
Current smoker	2 (13.3%)	6 (40.0%)			
Former smoker	2 (13.3%)	5 (33.3%)			
RF (*n*, %)		10 (66.7%)			
ACPA (*n*, %)		11 (73.3%)			
TJC		4.2 ± 7.0		3.1 ± 2.3	**0.041**
SJC		3.1 ± 1.8		0.9 ± 1.1	0.331
CRP (mg/dl)		1.89 ± 1.36		0.92 ± 0.68	0.234
ESR		26.6 ± 13.1		20.4 ± 10.4	0.591
PGA		6.4 ± 1.2		3.4 ± 1.4	0.063
PhGA		4.6 ± 0.9		2.5 ± 1.2	**0.046**
DAS28-CRP		4.61 ± 0.51		2.96 ± 0.7	**0.016**
DAS28-ESR		4.49 ± 0.72		3.15 ± 0.82	0.085
SDAI		23.3 ± 5.53		10.8 ± 5.8	**0.026**
CDAI		21.4 ± 5.1		9.9 ± 10.3	0.051
MTX dose				11.4 ± 0.8	
BMI	25.1 ± 1.8	24.7 ± 1.7	0.902		
QRISK-2 score	7.9 ± 10.2	18.9 ± 16.3	**0.029**		
Hypertension	4 (26.7%)	5 (33.3%)	0.5		
Diabetes mellitus	0	0	1.0		
Hypercholesterolaemia	4 (26.7%)	7 (46.7%)	0.449		
Hypertrygliceridaemia	2 (13.3%)	3 (20.0%)	0.5		

Data are shown as mean ± standard error or number (percentage)

### Angiogenic T cells

**Fig. 1:**
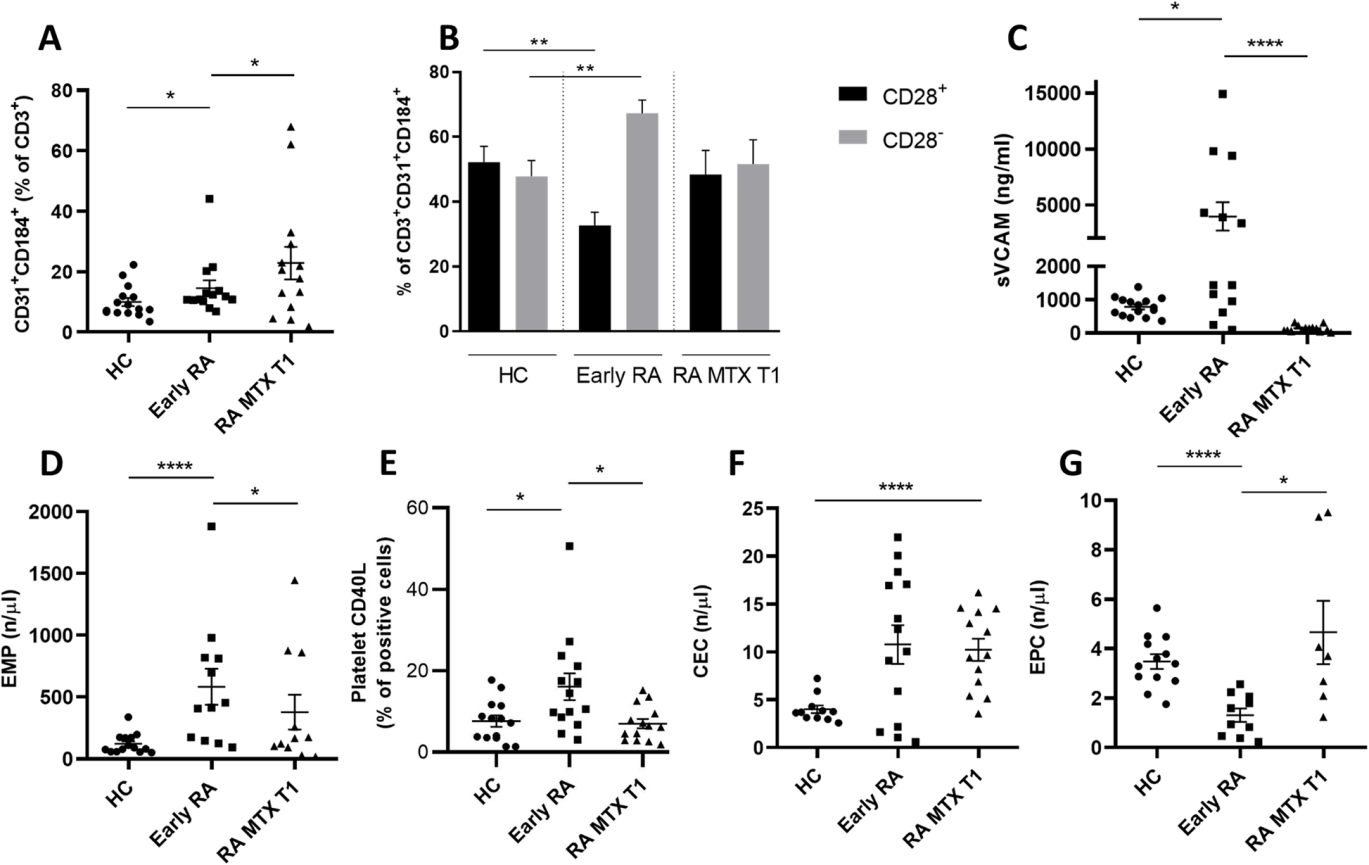
Effects of methotrexate on biomarkers of endothelial dysfunction. Circulating Tang cells are more prevalent in early RA compared to HCs, and their number further increases following MTX treatment (**A**). Early RA patients show an altered balance of Tang cells in favour of a CD28^null^phenotype which is restored following MTX treatment (**B**). In RA patients, 3-month MTX treatment restores sVCAM-1 (**C**), EMP (**D**), platelet CD40L (**E**) and EPC (**G**) to levels of HCs. CEC levels do not display significant differences (**F**). Data are shown as mean ± standard error. *p ≤ 0.05, **p ≤ 0.01, ***p ≤ 0.001, ****p ≤ 0.0001

At baseline, the percentage of Tang cells was significantly higher in RA patients as compared to HCs (14.6 ± 4.9% vs 10.0 ± 2.6%, respectively, *p* = 0.037). Interestingly, Tang cells significantly increased after 3 months of MTX treatment compared to baseline (22.9% ± 10.6% vs 14.6% ± 4.9%, *p* ≤ 0.03) (Fig. 1A). In order to analyse the specific Tang cell phenotype involved in both subject cohorts, we subsequently investigated the expression of surface CD28 on Tang cells. At baseline, Tang in RA patients were characterized by a higher proportion of CD28^null^cells (69.4 ± 8.6% vs 47.8 ± 9.5%, respectively, *p* = 0.003) and lower CD28-positive (32.7 ± 8.0% vs 52.2 ± 9.5%, respectively, *p* = 0.006) cells compared with HCs, showing an imbalance towards a CD28^null^Tang cell population. Of interest, 3 months of MTX therapy restored a Tang cell phenotype similar to that of HCs (Fig. 1B).

### Endothelial activation markers

In naïve RA patients, QRISK-2 score positively correlated with PMPs (rho = 0.657, *p* = 0.02), EPCs (rho = 0.673, *p* = 0.033) and CECs (rho = 0.569, *p* = 0.034). No significant correlation of QRISK-2 was found with Tang cells.

Subsequently, we evaluated the effect of MTX therapy on markers of endothelial dysfunction. At baseline, early RA patients displayed higher levels of sVCAM-1 compared to HCs (3973 ± 2410 vs 785 ± 283 ng/ml, respectively, *p* = 0.0188), which was significantly and markedly reduced after treatment with MTX (140 ± 49 ng/ml, *p* < 0.0001) (Fig. 1C). Similarly, at baseline, a significant higher concentration of circulating EMPs, a well-known marker of endothelial damage, was observed in early RA patients in comparison with HCs (583 ± 287/µl vs 122 ± 42/µl, respectively, *p* < 0.0001), and levels significantly reduced following MTX treatment (377 ± 277/µl, *p* = 0.015) (Fig. 1D).

Platelet CD40L expression was 7.63 ± 1.37% in HC, 16.08 ± 3.28% in early RA patients and 6.99 ± 1.15% in RA patients following a 3-month treatment with MTX (*p* = 0.0459 RA vs HCs and *p* = 0.0148 post- vs pre-MTX) (Fig. 1E), with a trend similar to that of sVCAM-1.

As expected, an opposed behaviour was observed for circulating EPCs, a marker of endothelial layer restoration. As shown in Fig. 1F, EPC levels were significantly lower in early RA compared to HCs at baseline (1.3 ± 0.5/µl vs 3.5 ± 0.7/µl, respectively, *p* < 0.0001) and significantly increased to levels similar to HCs after MTX treatment (4.7 ± 2.4/µl, *p* = 0.0156).

**Fig. 2:**
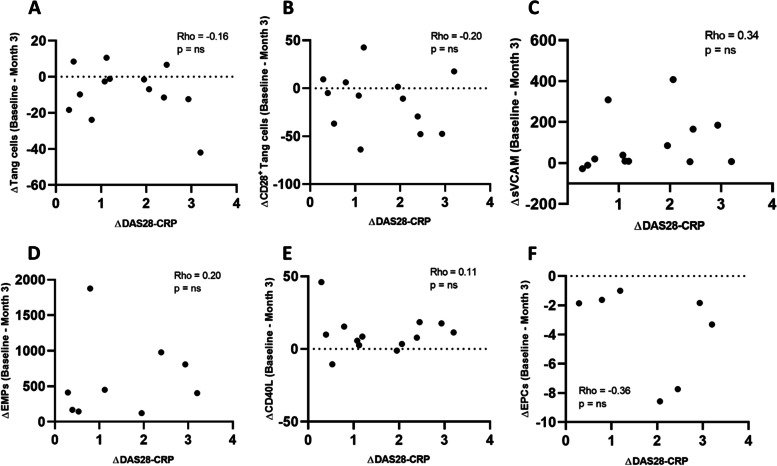
Correlation of endothelial dysfunction biomarkers changes with disease activity. The variation (Δ) of circulating Tang cells (**A**), CD28 + Tang cells (**B**), sVCAM-1 (**C**), EMPs (**D**), platelet CD40L (**E**) and EPCs (**F**) after 3 months of MTX treatment does not correlate with the change in disease activity according to DAS28-CRP

As far as CECs are concerned, we observed a trend towards higher levels of CECs in RA patients in comparison with HCs at baseline, although not statistically significant due to high variability (10.8 ± 3.9/µl vs 4.5 ± 1.6/µl, respectively, *p* = 0.077). MTX treatment did not seem to exert a significant effect on CEC levels (Fig. 1G). To evaluate whether the observed effects were attributable to direct MTX action, to the reduction of disease activity following treatment or to a combination of the two elements, we performed a correlation analysis of the Δ variation of DAS28-CRP between baseline and 3 months follow-up and the Δ variation of biomarkers of interest. No significant correlation was found between the Δ disease activity and any of the variables analysed (Fig. 2).

Finally, in order to investigate whether the beneficial effects on endothelial function were maintained beyond 3 months of MTX treatment, a cohort of fifteen sex- and age-matched RA patients (mean age 51.5 ± 2.7 years) treated with MTX (mean dosage 12.3 ± 0.8 mg/week) for more than 6 months were enrolled. The mean MTX treatment duration in this cohort was 29 ± 12.5 months. As shown in Table 2, the levels of Tang cells, CD28-positive and CD28^null^Tang cells as well as of platelet CD40L and of markers of endothelial damage and restoration, including EMPs, EPCs and CECs, were similar in both groups, thus suggesting a stable, long-lasting beneficial effect of MTX on endothelial homeostasis.

**Table 2:** Markers of endothelial dysfunction in early and long-standing MTX-treated RA patients

	**MTX at 3 months,** ***N* = 14**	**Long-standing MTX treatment,** ***N* = 15**	***p***
Tang (%)	22.9 ± 10.6	23.7 ± 6.0	0.533
CD28 + Tang (%)	48.4 ± 14.6	36.2 ± 9.2	0.146
CD28^null^Tang (%)	51.6 ± 14.6	63.8 ± 18.1	0.146
EMPs (*n*/µl)	377 ± 468	281 ± 155	0.919
Platelet CD40L (%)	6.9 ± 1.1	9.9 ± 1.9	0.201
EPCs (*n*/µl)	4.7 ± 2.5	6.7 ± 4.4	0.482
CECs (*n*/µl)	10.2 ± 2.3	14.9 ± 7.6	0.892

Markers of endothelial and platelet function do not significantly differ between RA patients treated with methotrexate for 3 months and patients with long-term treatment. Data are shown as mean ± standard error

## Discussion

Endothelial dysfunction represents one of the earliest triggers of atherosclerosis development, and it greatly contributes to the increased CV risk in patients with chronic inflammatory rheumatic diseases [1]. Thus, identifying treatment strategies able to improve or reverse endothelial damage represents an important and largely unmet need in these patients. Experimental evidence suggests that MTX, beyond its favourable effects on lipid profile and insulin resistance, may act directly on the endothelial layer by reducing inflammatory pathways and improving endothelial function. In the present study, we demonstrated that 3 months of MTX therapy exert a beneficial effect on cellular markers of endothelial damage and repair in a cohort of early RA patients and that these favourable activities persist during long-term treatment. To the best of our knowledge, this is the first demonstration of a favourable effect of MTX on the novel and clinically relevant Tang, EPCs and EMPs biomarkers in this population.

The contribution of Tang cells to the biology of the endothelium is well-established. Their main function is to orchestrate the activity of EPCs which are essential in the repair of endothelial injury [16]. Interestingly, as found in pSS [17] and anti-dsDNA-positive SLE patients [19], we demonstrated significantly higher baseline levels of circulating Tang cells in a cohort of early, treatment-naïve RA patients, notably free from previous major CV events. Thus, our cohort may not yet be characterized by a high level of endothelial damage and turnover as observed in patients with longer disease duration [14, 20] or with previous CV events which exhibit lower Tang levels [18, 20].

Moreover, we focused on the expression of CD28, the absence of which is typical in senescent CD4 + cells and characterizes RA patients with increased CV risk [38]. The absence of CD28 on Tang cells has been associated with the presence of other markers of senescence, such as CCR7, CD27 and CD57 [18, 22]. Of interest, CD4 + CD28^null^Tang cells, more prevalent in our RA cohort, have been associated with atherosclerotic risk in patients with hypertension [22]. In this setting, the ability of a 3-month MTX therapy to restore the imbalance between the two Tang cell subpopulations to levels similar to those of HCs may be one of the main mechanisms underlying the favourable effect of this drug on endothelial dysfunction.

Shedding of endothelial MPs is a marker of damage, stress and dysfunction. Increased circulating EMPs have been previously reported in RA [39], and our study confirms these findings. On the contrary, circulating EPCs are bone marrow-derived cells that, along with Tang cells, are closely involved in endothelial repair [40, 41]. In line with this hypothesis, we found higher levels of EMPs and lower levels of EPCs in RA subjects compared to HCs. Of note, MTX treatment was able to restore both parameters to levels not different from those of HCs. In addition, we also confirmed a strong effect of MTX in reducing circulating levels of sVCAM-1, along with platelet CD40L, important markers of endothelial dysfunction [4, 28, 42, 43]. On the contrary, CEC concentration, whose levels are expected to be higher in inflammatory conditions, was not significantly different between RA and HCs due to very high variability in the former group. Nevertheless, MTX treatment did not seem to exert any significant effect on this cell population.

The effects of MTX on the endothelium and how MTX reduces chronic inflammation in patients with inflammatory diseases remain poorly understood. Although some reports suggest a potential detrimental effect on the endothelium [44], recent studies are in line with our findings [26] and suggest a potential, early, immune-modulatory effect of MTX on specific cellular subsets involved in endothelial homeostasis impairment. In particular, MTX reduces TNF-α-induced endothelial cell activation by modulation of VCAM-1, ICAM-1 and E-Selectin expression. It is also able to inhibit endothelial cell apoptosis, to promote their proliferation and to reduce the release of pro-inflammatory cytokines such as IL-6 and MCP-1 [31, 45].

Moreover, the demonstration that in our study the favourable effects on endothelial homeostasis seem to be at least partly independent of the reduction of systemic inflammation fits well with this hypothesis and confirms the results of recent in vitro and population-based studies [24, 33]. In fact, in a cohort of patients with the CV risk profile, MTX therapy did not reduce the levels of some inflammatory cytokines, such as TNFα, IL-1β and IL-6 [46]. This entails that multiple, still unexplored mechanisms of action beyond the anti-inflammatory mechanisms of the drug may contribute to the favourable effect of MTX on endothelial function [29–33]. Moreover, increased levels of platelet CD40L in RA have been reported [47, 48], and it has been proposed as a potential therapeutic target [49]. In an adjuvant arthritis rat model, MTX administration significantly reduced CD40L expression in the whole blood [50]. Given that platelet CD40L is a key player of endothelial dysfunction [10], it can be also hypothesized that MTX-induced platelet CD40L decrease contributes to the observed amelioration of endothelial dysfunction.

Our study has some limitations. First, the effects of MTX on endothelium were evaluated at 3-month therapy only. Although studies with longer duration are needed, we confirmed the rapid effect of MTX in halting the progression of atherosclerosis [28, 33]. These effects may be more pronounced in patients with early disease, as our cohort, since a chronic inflammatory condition may induce progressive irreversible endothelial damage and exhaustion of reparative mechanisms [14]. However, the demonstration of similar levels of endothelial markers of damage and repair between patients treated for 3 months and a subsequently included cohort of age- and sex-matched RA subjects treated for at least 6 months suggests a potential long-lasting beneficial effect.

Secondly, the present study does not allow us to ascertain whether these effects are due to MTX treatment, reduced disease activity or both. Although in our RA cohort, disease activity at 3 months was very variable ranging from remission to high, the low number of subjects did not allow a sub-group analysis. Indeed, the positive effects on the endothelium demonstrated in patients treated with TNFα inhibitors suggest that suppression of inflammation itself may prevent atherosclerosis progression [26, 32]. However, in our study, the protective effect on the endothelium was, at least in part, independent of change in disease activity, suggesting a specific direct action of MTX.

## Conclusions

The results of the present study, although preliminary and to be confirmed by larger studies with longer follow-ups, confirm a rapid-onset beneficial effect of MTX on endothelial function in RA. In particular, we provide the first demonstration of its ability to restore a physiologic balance of circulating Tang cells, a population known to be directly involved in endothelial repair. This suggests that the rapid introduction of MTX therapy in RA patients with early disease may have additional CV benefits in RA patients independent of its role in controlling disease activity and systemic inflammation and further support its positive effects in reducing the risk of subsequent CV events in this population.

## Data Availability

The datasets used and/or analysed during the current study are available from the corresponding author upon reasonable request.

## Abbreviations

CAM: Cell adhesion molecule
CDAI: Clinical disease activity index
CEC: Circulating endothelial cell
CRP: C-reactive protein
CV: Cardiovascular
DAS28: Disease Activity Score on 28 Joints
ELISA: Enzyme-linked immunosorbent assay
EMP: Endothelial microparticle
EPC: Endothelial progenitor cell
ESR: Erythrocyte sedimentation rate
EV: Extracellular vesicles
FITC: Fluorescein isothiocyanate
HC: Healthy control
MTX: Methotrexate
PGA: Patient’s global assessment
PhGA: Physician’s global assessment
pSS: Primary Sjögren’s syndrome
RA: Rheumatoid arthritis
SDAI: Simplified Disease Activity Index
SJC: Swollen joint count
SLE: Systemic lupus erythematosus
sVCAM: Soluble vascular cell adhesion molecule
Tang: Angiogenic T cell
TJC: Tender joint count
TNF: Tumour necrosis factor

## References

[1] Botts SR, Fish JE, Howe KL (2021). Dysfunctional vascular endothelium as a driver of atherosclerosis: emerging insights into pathogenesis and treatment. Front Pharmacol.

[2] Falcinelli E, Francisci D, Belfiori B, Petito E, Guglielmini G, Malincarne L (2013). In vivo platelet activation and platelet hyperreactivity in abacavir-treated HIV-infected patients. Thromb Haemost.

[3] Falcinelli E, Cosmi B, Filippini M, Petito E, Legnani C, Cini M (2017). Endothelial activation in patients with superficial vein thrombosis (SVT) of the lower limbs. Thromb Res.

[4] Falcinelli E, Petito E, Becattini C, De Robertis E, Paliani U, Sebastiano M (2021). Role of endothelial dysfunction in the thrombotic complications of COVID-19 patients. J Infect.

[5] Francisci D, Falcinelli E, Belfiori B, Petito E, Fierro T, Baldelli F (2011). Impact of tenofovir versus abacavir on HIV-related endothelial dysfunction. AIDS Patient Care STDs.

[6] Francisci D, Falcinelli E, Baroncelli S, Petito E, Cecchini E, Weimer LE (2014). Potential anti-inflammatory effects of maraviroc in HIV-positive patients: a pilot study of inflammation, endothelial dysfunction, and coagulation markers. Scand J Infect Dis.

[7] Gresele P, Migliacci R, Vedovati MC, Ruffatti A, Becattini C, Facco M (2009). Patients with primary antiphospholipid antibody syndrome and without associated vascular risk factors present a normal endothelial function. Thromb Res.

[8] Hedar AM, Stradner MH, Roessler A, Goswami N (2021). Autoimmune rheumatic diseases and vascular function: the concept of autoimmune atherosclerosis. J Clin Med.

[9] Lauper K, Courvoisier DS, Chevallier P, Finckh A, Gabay C (2018). Incidence and prevalence of major adverse cardiovascular events in rheumatoid arthritis, psoriatic arthritis, and axial spondyloarthritis. Arthritis Care Res.

[10] Giannini S, Falcinelli E, Bury L, Guglielmini G, Rossi R, Momi S (2011). Interaction with damaged vessel wall in vivo in humans induces platelets to express CD40L resulting in endothelial activation with no effect of aspirin intake. Am J Physiol Heart Circ Physiol.

[11] André P, Nannizzi-Alaimo L, Prasad SK, Phillips DR (2002). Platelet-derived CD40L: the switch-hitting player of cardiovascular disease. Circulation.

[12] Cognasse F, Duchez AC, Audoux E, Ebermeyer T, Arthaud CA, Prier A (2022). Platelets as key factors in inflammation: focus on CD40L/CD40. Front Immunol.

[13] Lievens D, Zernecke A, Seijkens T, Soehnlein O, Beckers L, Munnix ICA (2010). Platelet CD40L mediates thrombotic and inflammatory processes in atherosclerosis. Blood.

[14] Bartoloni E, Alunno A, Bistoni O, Caterbi S, Luccioli F, Santoboni G (2015). Characterization of circulating endothelial microparticles and endothelial progenitor cells in primary Sjögren’s syndrome: new markers of chronic endothelial damage?. Rheumatol Oxf Engl.

[15] Farinacci M, Krahn T, Dinh W, Volk H-D, Düngen H-D, Wagner J (2019). Circulating endothelial cells as biomarker for cardiovascular diseases. Res Pract Thromb Haemost.

[16] Hur J, Yang H-M, Yoon C-H, Lee C-S, Park K-W, Kim J-H (2007). Identification of a novel role of T cells in postnatal vasculogenesis: characterization of endothelial progenitor cell colonies. Circulation.

[17] Alunno A, Ibba-Manneschi L, Bistoni O, Cipriani S, Topini F, Gerli R (2019). Angiogenic T cells in primary Sjögren’s syndrome: a double-edged sword?. Clin Exp Rheumatol.

[18] López P, Rodríguez-Carrio J, Martínez-Zapico A, Caminal-Montero L, Suarez A (2016). Senescent profile of angiogenic T cells from systemic lupus erythematosus patients. J Leukoc Biol.

[19] Miao J, Qiu F, Li T, Zhao P, Zhang K, Lv M (2016). Circulating angiogenic T cells and their subpopulations in patients with systemic lupus erythematosus. Mediators Inflamm.

[20] Rodríguez-Carrio J, Alperi-López M, López P, Alonso-Castro S, Ballina-García FJ, Suárez A (2015). Angiogenic T cells are decreased in rheumatoid arthritis patients. Ann Rheum Dis.

[21] de Boer SA, Reijrink M, Abdulahad WH, Hoekstra ES, Slart RHJA, Heerspink HJL (2020). Angiogenic T cells are decreased in people with type 2 diabetes mellitus and recruited by the dipeptidyl peptidase-4 inhibitor Linagliptin: a subanalysis from a randomized, placebo-controlled trial (RELEASE study). Diabetes Obes Metab.

[22] Zhang G, Liu Y, Qiu Y, Zhang J, Sun J, Zhou Z (2021). Circulating senescent angiogenic T cells are linked with endothelial dysfunction and systemic inflammation in hypertension. J Hypertens.

[23] Bălănescu A-R, Bojincă VC, Bojincă M, Donisan T, Bălănescu SM (2019). Cardiovascular effects of methotrexate in immune-mediated inflammatory diseases. Exp Ther Med.

[24] Johnson TM, Sayles HR, Baker JF, George MD, Roul P, Zheng C (2021). Investigating changes in disease activity as a mediator of cardiovascular risk reduction with methotrexate use in rheumatoid arthritis. Ann Rheum Dis.

[25] Roubille C, Richer V, Starnino T, McCourt C, McFarlane A, Fleming P (2015). The effects of tumour necrosis factor inhibitors, methotrexate, non-steroidal anti-inflammatory drugs and corticosteroids on cardiovascular events in rheumatoid arthritis, psoriasis and psoriatic arthritis: a systematic review and meta-analysis. Ann Rheum Dis.

[26] Deyab G, Hokstad I, Whist JE, Smastuen MC, Agewall S, Lyberg T (2017). Methotrexate and anti-tumor necrosis factor treatment improves endothelial function in patients with inflammatory arthritis. Arthritis Res Ther.

[27] Verhoeven F, Prati C, Chouk M, Demougeot C, Wendling D (2021). Methotrexate and cardiovascular risk in rheumatic diseases: a comprehensive review. Expert Rev Clin Pharmacol.

[28] Liu D, Lv H, Liu Q, Sun Y, Hou S, Zhang L (2019). Atheroprotective effects of methotrexate via the inhibition of YAP/TAZ under disturbed flow. J Transl Med.

[29] Ma Y, Li L, Shao Y, Bai X, Bai T, Huang X (2017). Methotrexate improves perivascular adipose tissue/endothelial dysfunction via activation of AMPK/eNOS pathway. Mol Med Rep.

[30] Thornton CC, Al-Rashed F, Calay D, Birdsey GM, Bauer A, Mylroie H (2016). Methotrexate-mediated activation of an AMPK-CREB-dependent pathway: a novel mechanism for vascular protection in chronic systemic inflammation. Ann Rheum Dis.

[31] Yang D, Haemmig S, Zhou H, Pérez-Cremades D, Sun X, Chen L (2021). Methotrexate attenuates vascular inflammation through an adenosine-microRNA-dependent pathway. eLife.

[32] Zălar D-M, Pop C, Buzdugan E, Kiss B, Ştefan M-G, Ghibu S (2021). Pharmacological effects of methotrexate and infliximab in a rats model of diet-induced dyslipidemia and beta-3 overexpression on endothelial cells. J Clin Med.

[33] Deyab G, Reine TM, Vuong TT, Jenssen T, Hjeltnes G, Agewall S (2021). Antirheumatic treatment is associated with reduced serum Syndecan-1 in rheumatoid arthritis. PLoS ONE.

[34] Aletaha D, Neogi T, Silman AJ, Funovits J, Felson DT, Bingham CO (2010). 2010 Rheumatoid arthritis classification criteria: an American College of Rheumatology/European League Against Rheumatism collaborative initiative. Arthritis Rheum.

[35] Mach F, Baigent C, Catapano AL, Koskinas KC, Casula M, Badimon L (2020). 2019 ESC/EAS Guidelines for the management of dyslipidaemias: lipid modification to reduce cardiovascular risk. Eur Heart J.

[36] Collins GS, Altman DG (2012). Predicting the 10 year risk of cardiovascular disease in the United Kingdom: independent and external validation of an updated version of QRISK2. BMJ.

[37] Gresele P, Migliacci R, Procacci A, De Monte P, Bonizzoni E (2007). Prevention by NCX 4016, a nitric oxide-donating aspirin, but not by aspirin, of the acute endothelial dysfunction induced by exercise in patients with intermittent claudication. Thromb Haemost.

[38] Gerli R, Schillaci G, Giordano A, Bocci EB, Bistoni O, Vaudo G (2004). CD4+CD28- T lymphocytes contribute to early atherosclerotic damage in rheumatoid arthritis patients. Circulation.

[39] Barbati C, Vomero M, Colasanti T, Ceccarelli F, Marcosano M, Miranda F (2018). Microparticles and autophagy: a new frontier in the understanding of atherosclerosis in rheumatoid arthritis. Immunol Res.

[40] van Zonneveld AJ, de Boer HC, van der Veer EP, Rabelink TJ (2010). Inflammation, vascular injury and repair in rheumatoid arthritis. Ann Rheum Dis.

[41] Westerweel PE, Verhaar MC (2009). Endothelial progenitor cell dysfunction in rheumatic disease. Nat Rev Rheumatol.

[42] Mangoni AA, Zinellu A, Sotgia S, Carru C, Piga M, Erre GL (2017). Protective effects of methotrexate against proatherosclerotic cytokines: a review of the evidence. Mediators Inflamm.

[43] Migliacci R, Guglielmini G, Busti C, Falcinelli E, Minuz P, Gresele P (2021). Walking-induced endothelial dysfunction predicts ischemic cardiovascular events in patients with intermittent claudication. Vasc Med Lond Engl.

[44] Merkle CJ, Moore IM, Penton BS, Torres BJ, Cueny RK, Schaeffer RC (2000). Methotrexate causes apoptosis in postmitotic endothelial cells. Biol Res Nurs.

[45] Liu X, Zhang R, Fu G, Sun Y, Wu J, Zhang M (2021). Methotrexate therapy promotes cell coverage and stability in in-stent neointima. Cardiovasc Drugs Ther.

[46] Ridker PM, Everett BM, Pradhan A, MacFadyen JG, Solomon DH, Zaharris E (2019). Low-dose methotrexate for the prevention of atherosclerotic events. N Engl J Med.

[47] Gasparyan AY, Stavropoulos-Kalinoglou A, Mikhailidis DP, Douglas KMJ, Kitas GD (2011). Platelet function in rheumatoid arthritis: arthritic and cardiovascular implications. Rheumatol Int.

[48] Habets KLL, Trouw LA, Levarht EWN, Korporaal SJA, Habets PAM, de Groot P (2015). Anti-citrullinated protein antibodies contribute to platelet activation in rheumatoid arthritis. Arthritis Res Ther.

[49] Karnell JL, Albulescu M, Drabic S, Wang L, Moate R, Baca M (2019). A CD40L-targeting protein reduces autoantibodies and improves disease activity in patients with autoimmunity. Sci Transl Med.

[50] Rui-Kai Z, Jian L (2012). Effects of xinfeng capsules on expression of platelet granule membrane protein 140 and platelet cluster of differentiation 40 ligand in peripheral blood of adjuvant arthritis rats. Int J Rheumatol.

